# The beginning of *Surgical Neurology International*

**DOI:** 10.4103/2152-7806.63896

**Published:** 2010-05-31

**Authors:** James I. Ausman

**Affiliations:** Editor-in-Chief, Surgical Neurology International

*Surgical Neurology International* (SNI) has begun publication of a free Internet journal of neurosurgery and neuroscience for the benefit of all 35,000 neurosurgeons around the world. This 21^st^ -century publication will not only publish scientific papers from around the world, but will have a Web site with free educational content for our readers in the developing and developed world.

The scientific papers will be published within 2 weeks of acceptance, making the publication time as short as possible. There will be no print versions, and therefore no delay in publishing. SNI will have papers on Translational Neuroscience for neurosurgeons, papers on new and innovative discoveries, and articles on Fundamental Neurosurgery and Neurology for our readers everywhere.

The Web site will contain a series of 100 lectures on all aspects of neurosurgery from the University of California, Los Angeles (UCLA) Department of Neurosurgery, lectures from other centers across the globe, a “Neurology for the Neurosurgeon” series, a Journal Club, Resident's Corner “How I Do It” videos for those in the developing world by Atos de Sousa from Brazil, and a case feedback blog. There will also be a section called Neurosurgery 2.0, using technology of the future in educating today's neurosurgeons worldwide. A neurosurgery Worldwide Market for exchange of needed equipment and people will start with the next issue.

There will be the expected popular editorials on socioeconomic, political, ethical and scientific issues, as well as invited editorials from experts in other fields of interest.

*Surgical Neurology International* is an independent journal with no affiliation to any organization. We are open to opinions of all kinds that reflect what neurosurgeons and scientists believe worldwide. We are also open to all new ideas you may have so as to provide information to your colleagues worldwide. We are interested in controversy and the truth that gets revealed as a result of discussion of different ideas. *Surgical Neurology International* is apolitical and recognizes the common bond all of us have as scientists seeking scientific truth and new ideas for the patients we treat.

Authors of accepted papers are charged a small fee for publication, which can be waived by the editor, if appropriate. With advertising and other revenues, we hope to offset that charge. There is no cost for downloading papers.

We are looking to involve young neurosurgeons all over the world who have great ideas that need to be seen, heard and read. If you are interested in becoming involved with this 21^st^ -century venture in information on neurosurgery, please write to us at editor@surgicalneurologyint.com.

We welcome suggestions about how we can provide content that will satisfy your needs.

If you would like to have our “Table of Contents” e-mailed to you each month, simply direct your Internet browser to http://www.surgicalneurologyint.com/signup.asp and complete the online form.


Sincerely yours,
James I. Ausman, MD, PhD

